# Common data elements for observational studies in ocular toxoplasmosis: a Delphi consensus

**DOI:** 10.1186/s12348-025-00525-2

**Published:** 2025-09-24

**Authors:** William Rojas-Carabali, Carlos Cifuentes-González, Kerry Goetz, Maria Vittoria Cicinelli, Zheng Xian Thng, Sally L. Baxter, Edmund Tsui, Padmamalini Mahendradas, Jyotirmay Biswas, Sofia Androudi, Andre Luiz Land Curi, Su Ling HO, Alfredo Adán, Rina La Distia Nora, Claudio Silveira, Heloisa Nascimento, João M. Furtado, Cristina Muccioli, Germán Mejía-Salgado, Cristhian A. Urzua, Justus G. Garweg, Ariel Schlaen, Xin Wei, Sivaraman Balamurugan, Ranju Kharel Sitaula, Ikhwanuliman Putera, Marcelo Rudzinski, Kalpana Babu, Mark Westcott, Rubens Belfort, Justine R. Smith, Jorge Gomez-Marin, Quan Dong Nguyen, Vishali Gupta, Rupesh Agrawal, Alejandra de-la-Torre

**Affiliations:** 1 Programme for Ocular Inflammation & Infection Translational Research, Department of Ophthalmology, National Healthcare Group Eye Institute, Tan Tock Seng Hospital, Singapore, Singapore; 2 Department of Ophthalmology, Tan Tock Seng Hospital, National Healthcare Group Eye Institute, Singapore, Singapore; 3 Lee Kong Chian School of Medicine, Nanyang Technological University, Singapore, Singapore; 4 Office of Data Science and Health Informatics, National Eye Institute, National Institutes of Health, Bethesda, MD USA; 5 School of Medicine, Vita-Salute San Raffaele University, Milan, Italy; 6 Department of Ophthalmology, IRCCS San Raffaele Scientific Institute, Milan, Italy; 7 Division of Ophthalmology Informatics and Data Science, Department of Ophthalmology and Shiley Eye Institute, Viterbi Family, University of California, La Jolla, San Diego, CA USA; 8 Division of Biomedical Informatics, Department of Medicine, University of California, La Jolla, San Diego, CA USA; 9 UCLA Stein Eye Institute, David Geffen School of Medicine at UCLA, Los Angeles, CA USA; 10 Department of Uveitis and Ocular Immunology, Narayana Nethralaya, Bengaluru, India; 11 Director of Uveitis & Ocular Pathology Department, Sankara Nethralaya, Chennai, India; 12 Department of Ophthalmology, University of Thessaly, Volos, Greece; 13 Infectious Ophthalmology Laboratory, Evandro Chagas National Institute of Infectious Diseases-FIOCRUZ, Rio de Janeiro, Brazil; 14 Hospital Clinic of Barcelona, Barcelona, Spain; 15 Department of Ophthalmology, Faculty of Medicine, University of Indonesia - Dr. Cipto Mangunkusumo Hospital, Jakarta, Indonesia; 16 Clínica Silveira, Erechim, Rio Grande do Sul Brazil; 17 Federal University of São Paulo, São Paulo, Brazil; 18 Division of Ophthalmology, Ribeirão Preto Medical School, University of São Paulo, Ribeirão Preto, São Paulo, Brazil; 19 Neuroscience (NEUROS) Research Group, Neurovitae Center for Neuroscience, Institute of Translational Medicine (IMT), Escuela de Medicina y Ciencias de la Salud, Universidad del Rosario, Bogotá, Colombia; 20 Centre of Excellence in Ocular Inflammation, Colombian Visual Science and Translational Eye Research Institute (CERI), Bogotá, Colombia; 21 Health Sciences Faculty, Universidad Autónoma de Bucaramanga UNAB, Bucaramanga, Colombia; 22 Laboratory of Ocular and Systemic Autoimmune Diseases, Faculty of Medicine, University of Chile, Santiago, Chile; 23 Facultad de Medicina, Clínica Alemana- Universidad del Desarrollo, Santiago, Chile; 24 Red de Investigación en Inmunología Ocular de Latinoamerica (RIOLAT), Santiago, Chile; 25 Swiss Eye Institute, Bern, Rotkreuz Switzerland; 26 Hospital de Clínicas “José de San Martín”, University of Buenos Aires/Hospital Universitario Austral, Buenos Aires, Argentina; 27 Department of Uveitis and Ocular Inflammation, Aravind Eye Hospital, Thavalakuppam, Pondicherry India; 28 Department of Ophthalmology, B. P. Koirala Lions Centre for Ophthalmic Studies, Institute of Medicine, Tribhuvan University, Kathmandu, Nepal; 29 Department of Ophthalmology and Department of Internal Medicine Section Clinical Immunology, Erasmus University Medical Center, Rotterdam, The Netherlands; 30 Universidad Católica de las Misiones, Posadas, Argentina; 31 Prabha Eye Clinic & Research Centre, Vittala International Institute of Ophthalmology, Bengaluru, India; 32 Barts Health NHS Foundation Trust, London, UK; 33 Department of Ophthalmology, University of Pittsburgh, Pittsburgh, USA; 34 Moorfields Eye Hospital NHS Foundation Trust, Uveitis & Scleritis Service, London, UK; 35 College of Medicine & Public Health, Flinders Health & Medical Research Institute, Flinders University, Adelaide, Australia; 36 Grupo GEPAMOL, Facultad de Ciencias de la Salud, Universidad del Quindío, Armenia, Colombia; 37 Byers Eye Institute, Stanford University, Palo Alto, CA USA; 38 Post Graduate Institute of Medical Education and Research (PGIMER), Advance Eye Centre, Chandigarh, India; 39 Singapore Eye Research Institute, Singapore, Singapore; 40 Duke NUS Medical School, Singapore, Singapore

**Keywords:** Ocular toxoplasmosis, Common data elements, Delphi consensus, Standardization, Clinical research, Uveitis

## Abstract

**Purpose:**

Ocular toxoplasmosis (OT) is the most common cause of posterior uveitis globally, with a significant risk of visual impairment. However, the lack of standardized data collection hinders meaningful comparisons across studies. This study aimed to develop a consensus-based set of Common Data Elements (CDEs) for observational studies in OT using a Delphi approach.

**Design:**

A set of CDEs was developed through a combination of a comprehensive literature review, a hybrid workshop, and a Delphi consensus process. This effort was led by an international panel of experts in OT to define a standardized CDE set for research and clinical purposes.

**Methods:**

A multidisciplinary steering committee identified an initial list of candidate CDEs through a targeted literature review. A panel of 30 international experts participated in a structured, one-round Delphi process to evaluate and refine these CDEs. Consensus was determined based on predefined thresholds for inclusion, exclusion, and modification.

**Results:**

A total of 139 CDEs were categorized across nine domains: Demographic and Background Information, Medical and Ocular History, Clinical Presentation, Clinical Findings, Lesion Characteristics, Diagnostics, Imaging Findings, Treatment and Interventions, and Outcomes. All 139 CDEs met the inclusion criteria, with 79.8% rated as “very important”. The consensus underscores the importance of a comprehensive, standardized dataset for OT research.

**Conclusions:**

This study establishes the first expert-derived standardized dataset requested for reporting OT outcomes, providing a framework to standardize data collection for future observational studies. Adopting these CDEs will enhance data comparability, improve meta-analyses, and strengthen the evidence base for clinical decision-making in OT. Future work will focus on real-world validation and refinement of this dataset.

**Supplementary Information:**

The online version contains supplementary material available at 10.1186/s12348-025-00525-2.

## Introduction

Ocular toxoplasmosis (OT), caused by the protozoan *Toxoplasma gondii*, is the most frequent cause of infectious posterior uveitis worldwide. It typically presents as focal retinochoroiditis, which can recur and progressively damage the retina, often leading to significant visual morbidity [1–4]. Notably, up to 35% of individuals with OT experience visual impairment (VI), with 20% suffering from blindness; [5] in some populations, VI prevalence reaches as high as 62% [6]. Beyond its ocular manifestations, toxoplasmosis is a global public health concern, with a high prevalence (up to 33%) that varies widely by region and is influenced by factors such as diet, environmental exposure, and immune status [2]. 

Despite substantial advances in diagnostic techniques and treatment strategies, OT remains challenging to manage due to its relapsing nature and diverse clinical presentations [7]. A key difficulty in advancing OT clinical research is the heterogeneity of outcome assessment across studies, which to some extent, can be explained by the variation in strain types and acquisition routes [8]. This variability hinders meaningful comparisons, complicates meta-analyses, and slows the development of robust, evidence-based clinical guidelines [5, 9]. 

To address this challenge, the concept of common data elements (CDEs) has gained interest in various fields, particularly those supported by the National Institutes of Health (NIH) [10, 11]. A CDE is a defined question paired with a set of permissible responses consistently used across multiple studies or projects to ensure uniform data collection and enhance data interoperability and reproducibility, particularly in biomedical research. CDEs provide a standardized framework for data collection, ensuring consistency in how variables—such as clinical signs, laboratory tests, and imaging findings—are defined and recorded [12]. In neurological research, for instance, the National Institute of Neurological Disorders and Stroke at the NIH has successfully promoted the adoption of CDEs to facilitate multi-center collaborations and accelerate advancements in patient care [10, 11]. By reducing variability in reporting and streamlining data harmonization, CDEs enhance the ability of researchers to pool data, conduct high-quality analyses, and generate stronger evidence to inform clinical practice [12]. 

Building on this approach, we aimed to establish a set of CDEs specifically tailored to OT, encompassing clinical assessments, imaging parameters, laboratory results, and patient-reported outcomes. These recommendations are designed to standardize and optimize data collection across diverse clinical settings and shorten the time needed for study design. By unifying how information is gathered, the proposed CDEs can advance our understanding of the disease course, improve the design and comparability of clinical studies, and ultimately guide more effective treatment strategies.

## Methods

This consensus approach was designed to gather expert input systematically and transparently, which is in line with current recommendations for conducting and reporting Delphi-based consensus studies.

### Selection of steering committee and participants

The consensus exercise was led by the Programme for Ocular Inflammation & Infection Translational Research (PROTON) [13], a platform of multicentric interdisciplinary collaboration aimed at confronting the complexities of ocular inflammatory and infectious diseases (OIID) (See protonstudy.org). The core steering committee (SC) comprised seven members—five uveitis specialists, one epidemiologist, and one data scientist—as well as two advisors: an ophthalmologist with dual board certifications in Ophthalmology and Clinical Informatics, and a representative from the National Eye Institute’s Office of Data Science and Health Informatics. The SC was formed by invitation based on professional reputation, publication record, and experience managing OT. The SC oversaw all aspects of the consensus process, including materials development, data analysis, and communication with the broader panel.

Participants were selected using purposive sampling to ensure representation from diverse clinical and geographic backgrounds. Invitations were extended to practicing ophthalmologists, uveitis specialists, and researchers with at least five years of clinical experience in OT or demonstrated expertise in ocular infectious disease. The SC aimed to include at least 25 participants to capture a broad range of opinions. A total of 30 participants were ultimately recruited through email invitations sent by the SC; no public advertisements or open calls were used. Participants were encouraged to recommend additional experts if they felt critical perspectives were missing. Patients and caregivers were not directly involved in the consensus development stage.

### Preparatory research

Before initiating the Delphi process, the SC conducted a targeted literature review in Web of Science (WoS) to identify existing definitions, diagnostic criteria, clinical endpoints, and outcome measures relevant to OT. The search retrieved 1,098 articles, with the top 100 cited studies yielding 63 for review. A refined search for the most updated research (2014–2024) retrieved 471 results. (Table 1) Studies were reviewed if they reported on clinical variables, outcomes, or classifications relevant to OT. Exclusion criteria included lack of OT-specific data or use of non-clinical models. Quality assessment was not performed for the reviewed studies. Additional information from published guidelines and expert opinion (including Utrecht Study Data Sheet) was also integrated into the initial list of 139 candidate CDEs presented for evaluation.

**Table 1:** Results of the targeted literature review in web of science

Search	Results	Screened*	Reviewed
“Ocular Toxoplasmosis” (All fields) and Article (Document Type) https://www.webofscience.com/wos/woscc/summary/5b27d6e2-816d-4d56-b825-2826740d06b6-01135a2491/times-cited-descending/1	1,098	Top 100 cited	63
“Ocular Toxoplasmosis” (All fields) and Article (Document Type) *Timespan: 2014-01-01 to 2024-10-18 (Publication Date)* https://www.webofscience.com/wos/woscc/summary/ea36c7ba-2362-488d-bf36-4d329b2b9c2a-01135b17ea/times-cited-descending/1	471	471	94

*Screening was made based on key terms “Epidemiology”, “Outcomes”, “Diagnosis”, “Clinical features”, “Clinical presentation”, “Treatment”. Case reports were excluded

Additional searches were conducted in the NIH, the EU Science Hub, and PubMed, using terms such as “ocular toxoplasmosis,” “uveitis,” “retinochoroiditis,” and “common data elements.” (Table 2) No relevant data were found in the NIH and EU Science Hub databases. PubMed returned only two articles, neither of which met the inclusion criteria. (Table 2) Given the lack of published literature on CDEs for ocular toxoplasmosis, a formal systematic review was deemed unfeasible; however, key references identified through this search were collated and shared with participants as background reading.

**Table 2:** Results of additional database searches for common data elements in OT

Source	Results
**National Institute of Health**,** USA** https://cde.nlm.nih.gov/home	0
**EU Science Hub** https://joint-research-centre.ec.europa.eu/index_en	0
**Pubmed** (“common data elements“[MeSH Terms] OR (“common“[All Fields] AND “data“[All Fields] AND “elements“[All Fields]) OR “common data elements“[All Fields]) AND (“toxoplasmosis, ocular“[MeSH Terms] OR (“toxoplasmosis“[All Fields] AND “ocular“[All Fields]) OR “ocular toxoplasmosis“[All Fields] OR (“ocular“[All Fields] AND “toxoplasmosis“[All Fields]))	Total results = 2 After screening = 0 (None was relevant)

After completing the preparatory research, a dedicated workshop entitled *“Ocular Toxoplasmosis Workshop: Towards a Common Data Model”* was conducted in the Centre for Healthcare Innovation, Singapore (24th Nov 2024). The event was recorded, and the recording was made available to all participants to ensure uniform access to the workshop content. During this workshop, experts presented a range of topics, including the clinical presentation and diagnosis of ocular toxoplasmosis, immunological responses and pathophysiology, and recent advances in treatment and management. The concept and relevance of developing CDEs were also introduced, and the proposed methodology for reaching consensus was discussed in detail. Feedback from the workshop participants was collated and used to refine the consensus approach before initiating the formal Delphi process.

### Assessing consensus

Numerous variations of the Delphi method have been introduced, yet its fundamental principles persist, including ensuring anonymity among participants and employing an organized feedback process carried out over the necessary rounds to achieve consensus [14, 15]. A modified Delphi approach was used to determine which candidate CDE warrants inclusion in a standardized OT set. For each potential CDE, participants were asked: “How would you rate the importance of collecting this variable in the dataset on ocular toxoplasmosis, and should it be collected in its current form or with modifications?”

They were provided with a single-response list of options:

1. Very Important – Collect as is
2. Very Important – Collect with modifications
3. Important – Collect as is
4. Important – Collect with modifications
5. Moderately Important – Collect as is
6. Moderately Important – Collect with modifications
7. Not Important – Do not collect
8. Not sure/No opinion

**Fig. 1:**
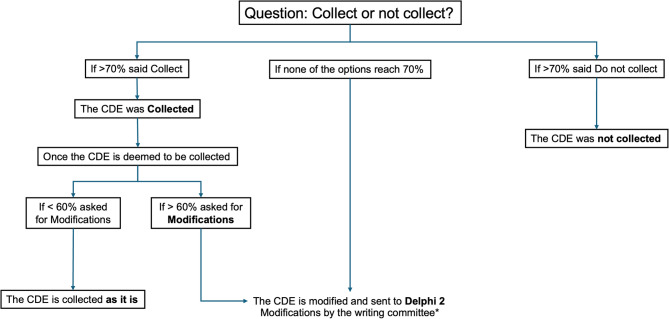
Flow diagram for the consensus rationale. If more than 70% of participants agree, the decision follows accordingly: if > 70% favor collection, CDE was collected; if > 70% oppose, CDE was not collected. Further modifications are considered when an agreement is below 70% in either direction. If proposed modifications receive less than 60% agreement, data is collected as is. If modifications receive more than 60% agreement, a second Delphi round (Delphi 2) was planned, incorporating additional refinements made by the writing committee, but it was not required

This combined rating scheme simultaneously gauged each item’s perceived importance (Modified from Lange et al. [16] as: Very Important, Important, Moderately Important, or Not Important) and whether it required modifications. We implemented the following process (Fig. 1):

1. Initial Classification (Collect or Do Not Collect). After the first round of responses, items for which ≥ 70% of participants selected any “Collect” option were marked for inclusion, whereas those for which ≥ 70% selected “Not Important – Do not collect” were excluded. Items that did not meet the ≥ 70% threshold for either category were deemed inconclusive [15, 17].
2. Modifications. Inconclusive items moved into a “modifications” phase. The SC reviewed free-text comments and suggested revisions to improve clarity or specificity. These modified items were then presented for re-evaluation. The item was retained if < 60% of participants still requested changes in the second review. If ≥ 60% indicated further revisions were needed, the item was deferred to a second Delphi round (Delphi 2).
3. Second Delphi Round. A second round of Delphi was planned; participants were supposed to re-rate each item that remained inconclusive or required additional modifications. The same ≥ 70% consensus threshold (for inclusion vs. exclusion) was considered to finalize decisions on these items. Figure 1 illustrates the decision-making process for determining whether to collect or not collect the CDEs based on consensus percentages.

### Participation

Participants received personalized invitations and two reminder emails to encourage participation. No financial incentives were provided. The surveys were conducted in English. The SC itself did not vote on item inclusion; however, it was responsible for collating feedback and determining whether any newly proposed variables should be included in the subsequent rating round.

### Anonymity and feedback

All responses were collected anonymously through a secure online platform (Cognito Forms, Columbia, South Carolina). After each step, the SC aggregated and shared anonymized feedback with participants, including summary statistics (e.g., the percentage of respondents who endorsed each option) and representative comments. No formal piloting of the survey instrument was carried out; however, the SC performed an informal review for clarity. Participants received three email reminders for each round to optimize response rates.

### Ethics statement

This study was conducted in accordance with the ethical principles outlined in the Declaration of Helsinki and was reviewed and approved by the Institutional Review Board of the National Healthcare Group under ID 2020/00301.

## Results

A total of 30 out of 38 experts responded to the first Delphi round (response rate 78.9%). Of these, 43% were from South America, 70% were subspecialists in uveitis, 66% had more than 10 years of clinical experience, and 67% reported seeing at least 20 cases of ocular toxoplasmosis per year (Table 3). Together, they evaluated 139 candidate CDEs spanning nine predefined domains: Demographic and Background Information (5 variables), Medical and Ocular History (5 variables), Clinical Presentation (4 variables), Clinical Findings (23 variables), Lesion Characteristics (10 variables), Diagnostics (10 variables), Image Findings (4 variables), Treatment and Interventions (21 variables), and Outcomes (57 variables). Supplementary Material 1 contains a detailed description of each CDE.

**Table 3:** Demographics and clinical experience of survey participants

Variable	Response	Frequency	Percentage
In what region do you practice?	East Asia	1	3%
	Europe	5	17%
	North America	1	3%
	Oceania	1	3%
	South America	13	43%
	South Asia	9	30%
What is the primary focus of your clinical practice?	Combined uveitis (50%) and retina (50%)	6	20%
	Mostly retina (> 50%)	1	3%
	Mostly uveitis (> 50%)	21	70%
	Other, please specify:	2	7%
How many years have you been practicing post-fellowship?	< 5 years	7	23%
	> 20 years	13	43%
	10–20 years	7	23%
	5–10 years	3	10%
How many patients with ocular toxoplasmosis have you managed in the last year?	0–20 cases	10	33%
	21–40 cases	7	23%
	41–60 cases	5	17%
	61–80 cases	3	10%
	> 80 cases	5	17%

**Fig. 2:**
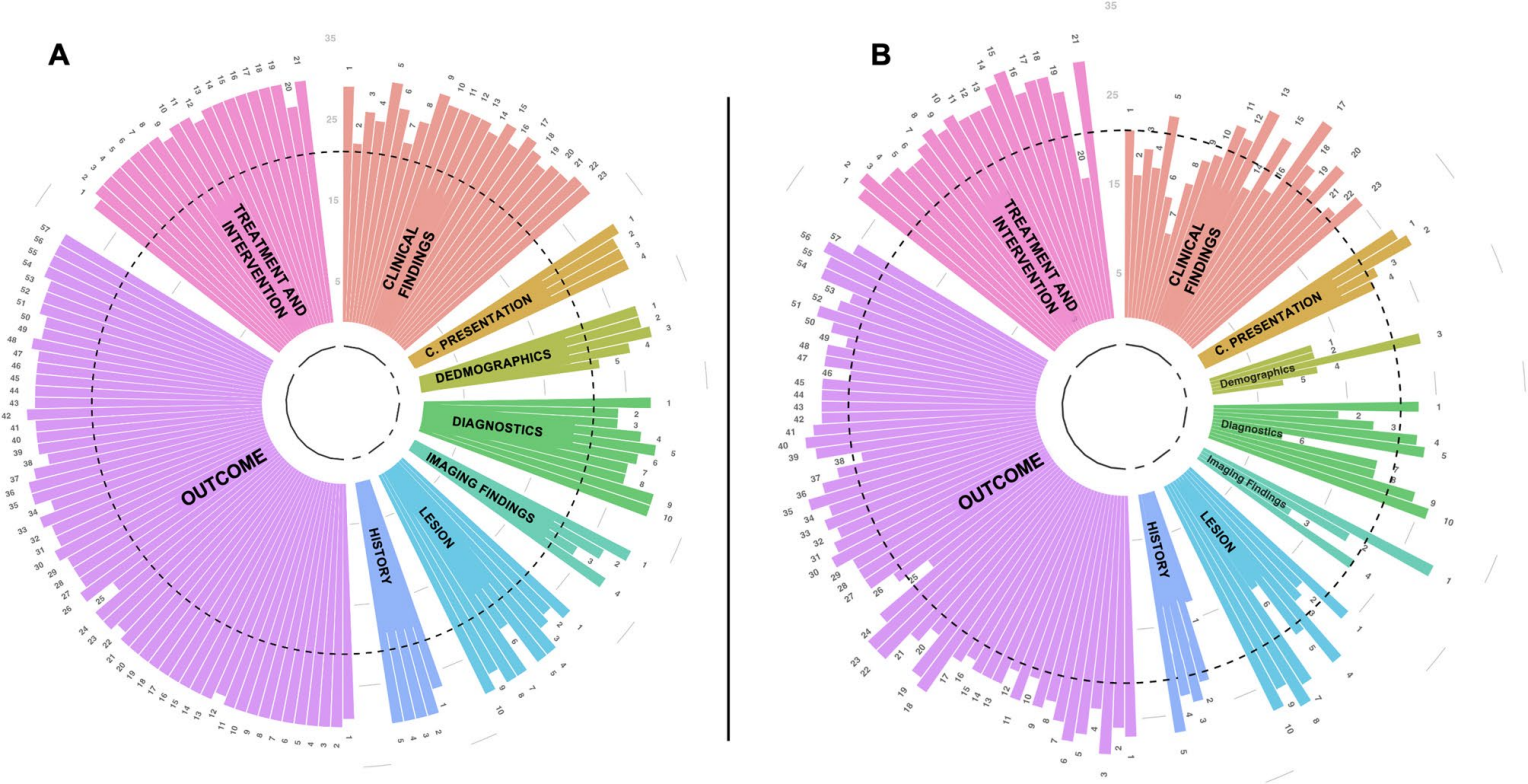
Frequency of responses across all Common Data Elements (CDEs). Each section represents a category of clinical or diagnostic variables, including outcomes, treatments and interventions, clinical findings, imaging results, and patient demographics. (A) shows the frequency of positive responses indicating the decision to collect each CDE, while (B) shows the frequency of positive responses considering each CDE as very important. The black dotted line represents the consensus threshold > 70%

All 139 CDEs reached the consensus threshold for inclusion without modifications (Fig. 2A). Therefore, modifications and Delphi round 2 were not required. Table 4 provides an illustrative overview of the findings for the Lesion Characteristics domain. Comprehensive results for each CDE, including expert consensus ratings, are available in Supplementary Material 2. Among them, 111 (79.8%) were rated as very important (Fig. 2B). This high level of consensus underscores the perceived value of collecting a comprehensive range of variables when investigating OT. The combined ratings indicate that while all CDEs are recognized as potentially valuable, further refinement or prioritization will likely depend on specific research objectives.

**Table 4:** Consensus results for the common data elements of the lesion characteristics domain

Data Element	Collect	Do not collect	As is	Modifications	Not sure/No opinion	Very important	Important	Moderately Important	Not important
Are the borders of the active lesion well-defined or irregular?	28 [93.3%]	1 [3.3%]	25 [89.3%]	3 [10.7%]	1 [3.3%]	24 [80.0%]	1 [3.3%]	3 [10.0%]	1 [3.3%]
Was Punctate outer retinal toxoplasmosis present at baseline?	27 [90.0%]	2 [6.7%]	26 [96.3%]	1 [3.7%]	1 [3.3%]	19 [63.3%]	5 [16.7%]	3 [10.0%]	2 [6.7%]
Was there any active retinochoroidal lesion present at the baseline visit?	30 [100.0%]	0 [0.0%]	25 [83.3%]	5 [16.7%]	0 [0.0%]	27 [90.0%]	3 [10.0%]	0 [0.0%]	0 [0.0%]
What are the characteristics of the inactive lesion?	30 [100.0%]	0 [0.0%]	26 [86.7%]	4 [13.3%]	0 [0.0%]	22 [73.3%]	5 [16.7%]	3 [10.0%]	0 [0.0%]
What is the shape of the active lesion?	24 [80.0%]	5 [16.7%]	23 [95.8%]	1 [4.2%]	1 [3.3%]	15 [50.0%]	6 [20.0%]	3 [10.0%]	5 [16.7%]
What is the size of the active lesion?	30 [100.0%]	0 [0.0%]	27 [90.0%]	3 [10.0%]	0 [0.0%]	27 [90.0%]	2 [6.7%]	1 [3.3%]	0 [0.0%]
What is the type of active lesion?	30 [100.0%]	0 [0.0%]	26 [86.7%]	4 [13.3%]	0 [0.0%]	29 [96.7%]	0 [0.0%]	1 [3.3%]	0 [0.0%]
What was the maximum number of lesions (including scars or active lesions from both eyes) observed at baseline?	28 [93.3%]	1 [3.3%]	26 [92.9%]	2 [7.1%]	1 [3.3%]	26 [86.7%]	2 [6.7%]	0 [0.0%]	1 [3.3%]
Where was the active lesion located?	30 [100.0%]	0 [0.0%]	27 [90.0%]	3 [10.0%]	0 [0.0%]	28 [93.3%]	1 [3.3%]	1 [3.3%]	0 [0.0%]
Did the patient present with features consistent with Acute Retinal Necrosis?	26 [86.7%]	2 [6.7%]	25 [96.2%]	1 [3.8%]	2 [6.7%]	18 [60.0%]	3 [10.0%]	5 [16.7%]	2 [6.7%]

Comprehensive results on expert consensus for each CDE are available in Supplementary Material 2

## Discussion

In this study, we employed a Delphi method to achieve expert consensus on a comprehensive set of CDEs for the study of OT. The high response rate among uveitis experts with extensive clinical experience strengthens the validity of our findings. Collectively, identifying and categorizing 139 CDEs—covering domains such as demographics, medical and ocular history, clinical presentation and findings, diagnostics, lesion characteristics, imaging findings, treatments, and outcomes—reflects OT’s complexity and multifaceted nature.

Our results demonstrate unanimous agreement that all 139 CDEs merit inclusion in a standardized CDE set, with nearly 80% rated *very important* (Table 5). These findings highlight a shared perception of their utility in guiding meaningful research and align with efforts in other medical fields to standardize data collection. Indeed, establishing standardized CDEs for OT provides an essential foundation for future research. By ensuring consistent data collection, these CDEs will facilitate comparability across studies, improving the ability to conduct meta-analyses and generate high-quality evidence for clinical guidelines [18]. Those CDEs rated as very important can serve as a core dataset for studies with limited resources, whereas the remaining CDEs can be used as optional variables depending on context, infrastructure, and study aims.

**Table 5:** List of the 111 common data elements rated as “very important” by expert consensus

Data Element	Definition	Allowable Responses
Did an epiretinal membrane (ERM) develop over the course of the disease?	Presence of a membrane formed on the retinal surface in association with the toxoplasmic lesion.	1 = Yes 0 = No 99 = Unknown
Did choroidal neovascularization (CNV) develop over the course of the disease?	Presence of new abnormal blood vessels growing from the choroid.	1 = Yes 0 = No 99 = Unknown
Did cystoid macular edema develop over the course of the disease?	Presence of cystoid macular edema.	1 = Yes 0 = No 99 = Unknown
Did posterior synechiae develop over the course of the disease?	Formation of adhesions between the iris and lens.	1 = Yes 0 = No 99 = Unknown
Did retinal arterial occlusion develop over the course of the disease?	Indicates whether the patient has been diagnosed with retinal arterial occlusion, a condition where the blood flow in one of the retinal arteries is blocked, potentially leading to vision loss.	1 = Yes 0 = No 99 = Unknown
Did retinal breaks develop over the course of the disease?	Indicates whether the patient has been diagnosed with retinal breaks	1 = Yes 0 = No 99 = Unknown
Did retinal detachment occur at any point during the disease progression?	Indicates whether the patient has been diagnosed with retinal detachment.	1 = Yes 0 = No 99 = Unknown
Did retinal ischemia develop over the course of the disease?	Indicates whether the patient has been diagnosed with ischemic retinal areas	1 = Yes 0 = No 99 = Unknown
Did retinal vein occlusion develop over the course of the disease?	Indicates whether the patient has been diagnosed with occlusion of the retinal veins.	1 = Yes 0 = No 99 = Unknown
Did the patient experience any side effects from the intravitreal injection for ocular toxoplasmosis, and if so, what were the primary side effects observed?	Records the side effects experienced by the patient from the treatment with intravitreal injection for ocular toxoplasmosis.	0 = None 1 = Irritation 2 = Subconjunctival hemorrhage 3 = Air bubble 4 = Corneal abrasion 5 = Iritis 6 = Vitreous hemorrhage 7 = Endophthalmitis 8 = Increased intraocular pressure 9 = Posterior vitreous detachment Conjunctivitis 10 = Hyphema 11 = Vitreous cell 12 = Conjunctival abrasion 13 = Medication in anterior chamber 14 = Corneal edema 15 = Retinal tear 16 = Rhegmatogenous retinal detachment 17 = central artery occlusion, 18 = exudative retinal detachment, 19 = Other 99 = Unknown
Did the patient experience any side effects from the treatment for ocular toxoplasmosis, and if so, what were the primary side effects observed?	Records any adverse effects experienced by the patient during treatment for ocular toxoplasmosis.	0 = No side effects (The patient did not experience any side effects) 1 = Gastrointestinal symptoms (e.g., nausea, vomiting, diarrhea) 2 = Skin rash (Allergic or non-allergic rash) 3 = Headache (Mild to severe headache) 4 = Fatigue (Generalized tiredness or lack of energy) 5 = Visual disturbances (Blurry vision or other vision-related side effects) 6 = Increased intraocular pressure (Elevation in eye pressure, often due to corticosteroid use) 7 = Liver function abnormalities (Detected by elevated liver enzymes, common with certain antibiotics) 8 = Bone marrow suppression (Reduced blood cell counts, potential side effect of antibiotics) 9 = Allergic reaction (General allergy symptoms such as itching, hives, or anaphylaxis) 10 = Renal function impairment (Impaired kidney function, common with certain medications) 11 = Mood changes or irritability (Emotional disturbances, sometimes associated with corticosteroids) 12 = Other (specify) (Other side effects not listed above) 99 = Unknown (It is unknown if the patient experienced side effects)
Has there been a recurrence of ocular toxoplasmosis during the follow-up?	Documentation of any recurrence of ocular toxoplasmosis during the follow up period.	1 = Yes 0 = No 99 = Unknown
How did the patient respond to treatment?	Refers to changes in lesion size or disease activity following treatment initiation, comparing the baseline characteristics and the latest follow up.	1 = Worsening (lesion enlarging or inflammation increasing), 2 = No improvement, 3 = Improvement with residual inflammation/sequale, 4 = Early recurrence (before 12 weeks) after treatment stop 5 = Recurrence after 12 weeks of treatment stop 5 = Complete resolution, 99 = Unknown
How long had been the patient under prophylaxis?	The total period of time during which prophylactic treatment was administered prior to the current episode.	Integer (months)
How long was the prophylaxis treatment administered?	The length of time the prophylactic treatment was administered to the patient, providing context for the preventive approach used.	Integer (months)
How many intravitreal injections of clindamycin plus dexamethasone were administered?	The total count of injections of of clindamycin plus dexamethasone administered directly into the eye’s vitreous cavity as part of treatment.	Integer
How many intravitreal injections of clindamycin without dexamethasone were administered?	The total count of injections of clindamycin without dexamethasone administered directly into the eye’s vitreous cavity as part of treatment.	Integer
How many times has the infection recurred?	The count of times the inflammation has returned after initial treatment.	Integer
How was the infection acquired?	The method or context in which the patient contracted the infection (e.g., congenital, postnatally, or undetermined).	1 = Congenital OT 2 = Postnatally acquired OT 3 = Undetermined 99 = Unknown
If recurrent, was any previous episode treated with antibiotics?	Indicates whether antibiotic treatment was administered during any prior episode related to the current condition. This helps assess previous treatment approaches and can influence current treatment decisions.	1 = Yes 0 = No 99 = Unknown
If the infection is congenital, is there evidence of a macular coloboma?	Indicates whether a macular coloboma is present in patients with a congenital condition. A macular coloboma is a structural defect in the macula, typically appearing as a missing piece or gap, and can significantly impact central vision.	1 = Yes, 0 = No, 99 = Unknown
On what date did the patient first experience side effects from the systemic treatment for ocular toxoplasmosis?	The date when the patient first reported or was observed to have side effects related to the systemic treatment for ocular toxoplasmosis.	Date (MM/DD/YYYY)
On what date did the patient first experience side effects from the intraocular injection?	The date when the patient first reported or was observed to have side effects related to the intraocular injection	Date (MM/DD/YYYY)
On what date was retinal scarring or evidence of healing first observed following the treatment of the active toxoplasmic lesion?	The specific date when retinal scarring or signs of healing were first documented after an active toxoplasmic lesion resolved.	Date (DD/MM/YYYY), Unknown
On what date was steroid therapy initiated?	Date of start of steorid therapy.	Date (DD/MM/YYYY).
On what date was steroid therapy stopped?	Date of end of steorid therapy.	Date (DD/MM/YYYY).
Recurrences: How many recurrences during the 2 years after first observed episode?	total number of recurrences of ocular toxoplasmosis observed in the two years following the first diagnosed episode.	Integer; 99 = unknown
Recurrences: How many recurrences during the 5 years after first observed episode?	total number of recurrences of ocular toxoplasmosis occurring within five years of the initial episode.	Integer; 99 = unknown
Was a Goldmann-Witmer test conducted, and what were the findings?	An antibody test used in ocular infections to detect specific antibodies within the eye, helping to confirm the presence of an infection like toxoplasmosis in the eye.	1 = Positive (> 3), 2 = Negative, 3 = Not done, 99 = Unknown
Was an avidity test performed, and if so, what were the results?	A diagnostic test measuring the strength of the IgG antibody binding to a specific antigen, used to determine the timing of infection (e.g., recent versus past infection).	1 = Weak, 2 = Strong, 0 = Not done 99 = Unknown
Was antibiotic treatment started, if active?	Indicates whether antibiotic therapy was initiated in response to an active lesion.	1 = Yes 0 = No 99 = Unknown
Was prophylaxis given to the patient?	Preventive treatment given to the patient to reduce the risk of infection or recurrence of the primary infection.	0 = No 1 = Yes, I give prophylaxis routinely 2 = Yes, due to multiple recurrences 3 = Yes, due to immunosuppresion 4 = Yes, due to lesion location 5 = Yes, due to recent acquired infection 99 = Unknown
Was the patient being treated with oral corticosteroid on the visit with the highest recorded vitreous humor *cells*, either eye?	Determines whether the patient was receiving oral corticosteroid treatment at the visit when the highest number of vitreous humor cells was recorded in either eye.	1 = Yes 0 = No 99 = Unknown
Was the patient under prophylaxys at baseline?	prophylaxys refers to preventive treatment aimed at reducing the risk of infection or recurrence in the patient.	1 = Yes 0 = No 99 = Unknown
Was there any active retinochoroidal lesion present at the baseline visit?	Indicates whether an active lesion was identified at the patient’s baseline visit.	1 = Yes, primary lesion without previous retinochoroidal scar, 2 = Yes, active lesion near to the border of a scar, 3 = Yes, active lesion far from the previos scars, 4 = Yes, light in teh fog image, 0 = No 99 = Unknown
Was there any evidence of coinfection?	The presence of an additional infection alongside the primary infection, which can impact disease severity, progression, and treatment needs.	1 = Yes 0 = No 99 = Unknown
Was there any subretinal fluid observed over the course of the disease?	Presence of subretinal fluid accumulation near or surrounding the lesion.	1 = Yes 0 = No 99 = Unknown
Was there any vascular changes at baseline?	Changes in the retinal vessels around or near the lesion clinically identified	1 = Normal vessels, 2 = Vessel sheathing, 3 = Keyrelis plaques, 4 = Arterial occlusion, 5 = Venous occlusion, 6 = Perivascular hemorrhages 7 = Retinal neovascularization 7 = Other, 99 = Unknown
Was there evidence of scleritis at baseline?	Presence of scleritis based on the clinical suspicion at baseline	0 = No, 1 = Yes, anterior scleritis, 2 = Yes, posterior scleritis, 3 = Yes, both (anterior and posterior scleritis), 99 = Unknown.
Was there resolution of the active lesion after treatment?	Evidence of scarring left/healing meaning resolution of an active toxoplasmic lesion after treatment.	1 = Yes 0 = No 2 = Partial 99 = Unknown
Was there segmental atrophy of the iris?	Presence of segmental atrophy of iris at baseline	1 = Yes 0 = No 99 = Unknown
Were all episodes treated with antiparasitic drug and corticosteroid?	whether every episode of ocular toxoplasmosis was managed using a combination of antiparasitic medication and corticosteroids.	1 = Yes 0 = No 99 = Unknown
Were any changes in the treatment plan required, and if so, what was the primary reason?	Indicates whether the treatment plan was modified during the course of care, with response options specifying both the occurrence and the primary reason for any required changes.	0 = No changes required 1 = Yes, due to lack of efficacy 2 = Yes, due to adverse effects 3 = Yes, due to disease progression 4 = Yes, due to patient preference or non-compliance 5 = Yes, due to availability of new treatment options 6 = Yes, other reason 99 = Unknown
Were any new retinochoroidal lesions documented during the follow-up period?	Documentation of new lesions during follow-up.	1 = Yes 0 = No 99 = Unknown
Were any surgical interventions performed as part of the treatment?	Surgical procedures performed for ocular toxoplasmosis complications.	1 = Yes 0 = No 99 = Unknown
Were iridic granulomas present at baseline?	Presence of iridic granulomas at baseline. Koeppe: at the pupil border. Bussaca: in the iris stroma	1 = Yes, Koeppe, 2 = Yes, Bussaca, 3 = Yes, both (Koeppe and Bussaca), 0 = No 99 = Unknown
Were keratic precipitates observed at baseline?	The presence of keratic precipitates at baseline and their characteristics	1 = Yes, mutton fat 2 = Yes, fine KPs 3 = Yes, both (mutton fat and fine) 0 = No 99 = Unknown
Were steroids started, either systemically or ocularly?	Use of corticosteroids for treatment.	1 = Yes (Systemic) 2 = Yes (Topical) 3 = Yes (Local intravitreal) 4 = Yes, (Local periocular) 3 = Yes, both (sytemic and topical/local), 0 = No 99 = Unknown
Were there any systemic symptoms present at baseline?	Indicates the presence of specific systemic symptoms (e.g., flu-like symptoms, lymphadenopathy) observed at the baseline visit.	0 = No systemic symptoms (Neither flu-like symptoms, lymphadenopathy, nor other systemic symptoms are present at baseline) 1 = Flu-like symptoms (Symptoms similar to influenza, such as fever, fatigue, body aches) 2 = Lymphadenopathy (Swollen or enlarged lymph nodes) 3 = Fever (Elevated body temperature indicating infection or inflammation) 4 = Rash (Skin rash, which may indicate systemic inflammation or infection) 5 = Fatigue (Generalized tiredness or lack of energy) 6 = Weight loss (Unintended weight loss suggesting a systemic process) 7 = Both flu-like symptoms and lymphadenopathy (Patient exhibits both flu-like symptoms and lymphadenopathy at baseline) 8 = Multiple symptoms (Patient exhibits more than two of the above symptoms at baseline; specify in notes if needed) 99 = Unknown (Information on baseline systemic symptoms is unavailable)
What antibiotic regimen was prescribed for the patient?	Specific antibiotics (anti toxoplasma agent) used for treatment.	1 = Trimethoprim–sulfamethoxazole, 2 = Clindamycin, 3 = Clindamycin (with dexamethasone), 4 = Pyrimethamine (with folinic acid), 5 = Azithromycin, 6 = Sulfadiazine, 7 = Spiramycin, 8 = Acetylspiramycin, 9 = Atovoquone, 10 = Other, 99 = Unknown
What are the characteristics of the inactive lesion?	Features that indicate an inactive lesion (If multiple, select the largest).	1 = Pigmented, 2 = Atrophic scar without pigmentary changes, 3 = Fibrosis, 4 = Combination of fibrosis and atrophy, 5 = Other. 99 = Uknown
What findings were observed on FA in the lesion during the active episode?	Characteristics of the lesion on fluorescein angiography during the active episode.	1 = None, 2 = Early lesion hyperfluorescence, 3 = Late lesion leakage, 4 = Lesion Staining, 5 = Lesion Blockage, 6 = Arterial Occlusion, 7 = Venous Occlusion, 8 = Vascular leakage and/or sheating, 9 = Optic disc leakage, 10 = Other, 99 = Unknown
What findings were observed on ICGA during the active episode?	Characteristics of the lesion on ICGA imaging.	1 = None, 2 = Hypofluorescence, 3 = Hyperfluorescence, 4 = Choroidal neovascularization, 5 = Other, 99 = Unknown
What is the patient’s city of origin?	The specific city where the patient was born or lived before their diagnosis, which may provide additional context related to regional health risks or environmental exposures.	
What is the patient’s country of origin?	The country where the patient was born or lived before their diagnosis, providing context for epidemiological, cultural, or genetic factors that may influence health	
What is the patient’s immune status?	The current health of the patient’s immune system, indicating its ability to respond to infections or disease.	1 = No immunosuppresed, 2 = HIV positive, 3 = Chronic oral corticosteroid therapy, 4 = Congenital immunodeficiency, 5 = Immunosuppressive drug therapy, 6 = Other adquired immunosuppresion (i.e. cancer), 7 = Pregnancy, 8 = Other 99 = Unknown.
What is the shape of the active lesion?	Shape or configuration of the active lesion (If multiple, select the largest).	1 = Round, 2 = Oval, 3 = Irregular, 4 = Crescent-shaped, 5 = Other, 99 = Unknown
What is the size of the active lesion?	Size of the active lesion in DD (If multiple, select the largest).	1 = < 1 DD, 2 = 1–2 DD, 3 = 2–3 DD, 4 = 3–4 DD 5 = > 4 DD, 99 = Unknown
What is the size of the largest lesion observed in either eye?	Records the size of the largest lesion, measured in millimeters or an equivalent unit, present in either eye.	1 = < 1 disc area (DA); 2 = 1-2DA; 3 = > 2DA, but ≤ 5% of fundus (comparable to area of macula); 4 = > 5% of fundus; 99 = unknown.
What is the type of the active lesion?	Type of active lesion.	1 = Unifocal well-circumscribed, 2 = Unifocal Diffuse, 3 = Multifocal 99 = Unknown
What prophylaxis regimen was being administered at baseline?	The specific medications, dosages, and schedule used in the prophylactic treatment.	1 = Trimethoprim–sulfamethoxazole, 2 = Clindamycin, 3 = Clindamycin (with dexamethasone), 4 = Pyrimethamine (with folinic acid), 5 = Azithromycin, 6 = Sulfadiazine, 7 = Spiramycin, 8 = Acetylspiramycin, 9 = Atovoquone, 10 = Other, 99 = Unknown
What type of infection does the patient have?	The specific classification of the infection affecting the patient (e.g., primary, recurrent, undetermined).	1 = Primary OT (no old scars in either eye) 2 = Recurrent OT (scars in either eye) 3 = Undetermined 99 = Unknown
What type of surgery was performed?	The specific surgical procedure performed as part of the patient’s treatment plan.	0 = None 1 = Vitrectomy (Pars Plana Vitrectomy) 2 = Retinal Detachment Repair 3 = Epiretinal Membrane Peeling 4 = Cataract Surgery 5 = Glaucoma Surgery 6 = Intravitreal Injection Port Placement 7 = Enucleation 8 = Other 99 = Unknown
What was the degree of anterior chamber inflammation at baseline?	Degree or severity of anterior chamber inflammation, using the SUN Criteria	1 = 0 (< 1 cell) 2 = 0.5+ (1–5 cells) 3 = 1+ (6–15 cells) 4 = 2+ (16–25 cells) 5 = 3+ (26–50 cells) 6 = 4+ (> 50 cells) 99 = Unknown
What was the clinical setting and level of care (e.g., primary, secondary, or tertiary) in which the patient received treatment?	Clinical Setting: The specific healthcare environment where the patient received treatment, including the level of care provided. This is often categorized into: Primary Care: The first point of contact for general health concerns, such as in a primary care physician’s office. Secondary Care: Specialized medical services, typically provided by a specialist or in a community hospital setting. Tertiary Care: Highly specialized, advanced care usually offered in a major hospital or medical center, such as for complex surgeries or specialized treatments.	1 = Primary, 2 = Secondary, 3 = Tertiary, 4 = Other, 99 = Unknown.
What was the date of the initial appearance or diagnosis of the arterial occlusion?	The specific date when the arterial occlusion was first observed or formally diagnosed in the patient.	Date (DD/MM/YYYY), Unknown
What was the date of the initial appearance or diagnosis of the cataract?	The specific date when the cataract was first observed or formally diagnosed in the patient.	Date (DD/MM/YYYY), Unknown
What was the date of the initial appearance or diagnosis of the choroidal neovascularization?	The specific date when the choroidal neovascularization was first observed or formally diagnosed in the patient.	Date (DD/MM/YYYY), Unknown
What was the date of the initial appearance or diagnosis of the cystoid macular edema?	The specific date when the cystoid macular edema was first observed or formally diagnosed in the patient.	Date (DD/MM/YYYY), Unknown
What was the date of the initial appearance or diagnosis of the epiretinal membrane?	The specific date when the epiretinal membrane was first observed or formally diagnosed in the patient.	Date (DD/MM/YYYY), Unknown
What was the date of the initial appearance or diagnosis of the ischemic retina?	The specific date when the ischemic retina was first observed or formally diagnosed in the patient.	Date (DD/MM/YYYY), Unknown
What was the date of the initial appearance or diagnosis of the optic nerve head atrophy?	The specific date when the optic nerve head atrophy was first observed or formally diagnosed in the patient.	Date (DD/MM/YYYY), Unknown
What was the date of the initial appearance or diagnosis of the phthisis?	The specific date when the phthisis was first observed or formally diagnosed in the patient.	Date (DD/MM/YYYY), Unknown
What was the date of the initial appearance or diagnosis of the posterior synechiae?	The specific date when the posterior synechiae was first observed or formally diagnosed in the patient.	Date (DD/MM/YYYY), Unknown
What was the date of the initial appearance or diagnosis of the retinal break?	The specific date when the retinal break was first observed or formally diagnosed in the patient.	Date (DD/MM/YYYY), Unknown
What was the date of the initial appearance or diagnosis of the retinal detachment?	The specific date when the retinal detachment was first observed or formally diagnosed in the patient.	Date (DD/MM/YYYY), Unknown
What was the date of the initial appearance or diagnosis of the subretinal fibrosis?	The specific date when the subretinal fibrosis was first observed or formally diagnosed in the patient.	Date (DD/MM/YYYY), Unknown
What was the date of the initial appearance or diagnosis of the subretinal fluid?	The specific date when the subretinal fluid was first observed or formally diagnosed in the patient.	Date (DD/MM/YYYY), Unknown
What was the date of the initial appearance or diagnosis of the uveitic glaucoma?	The specific date when the uveitic glaucoma was first observed or formally diagnosed in the patient.	Date (DD/MM/YYYY), Unknown
What was the date of the initial appearance or diagnosis of the vein occlusion?	The specific date when the retinal vein occlusion was first observed or formally diagnosed in the patient.	Date (DD/MM/YYYY), Unknown
What was the date of the initial appearance or diagnosis of the vitreous hemorrhage?	The specific date when the vitreous hemorrhage was first observed or formally diagnosed in the patient.	Date (DD/MM/YYYY), Unknown
What was the date of the last follow-up visit recorded?	Date of last follow-up visit	Date (DD/MM/YYYY), Unknown
What was the date of the patient’s baseline visit?	The date on which the patient’s baseline visit occurred, marking the starting point of formal assessments and observations in their treatment or study timeline.	Date (DD/MM/YYYY).
What was the date when ocular symptoms first began?	Date when ocular symptoms first appeared.	Date (DD/MM/YYYY).
What was the degree of vitreous **cells** developed at baseline?	Degree or severity of vitritis, using the following Criteria	1 = 0 (< 1 cell) 2 = 0.5+ (1–5 cells) 3 = 1+ (6–15 cells) 4 = 2+ (16–25 cells) 5 = 3+ (26–50 cells) 6 = 4+ (> 50 cells) 99 = Unknown
What was the degree of vitreous haze at baseline?	Degree or severity of inflammation of the vitreous, using the Vitreous Haze severity (NEI grading)	1 = 0 (Nil) 2 = 1 (Post pole clearly visible) 3 = 2 (Post pole details slightly hazy) 4 = 3 (Post pole details very hazy) 5 = 4 (Post pole details barely visible) 6 = 5 (Fundus details not visible) 99 = Unknown
What was the highest intraocular pressure recorded over the course of the disease?	Highest intraocular pressure recorded over the course of the disease	Integer (mmHg)
What was the interval between the first episode and the first subsequent recurrence?	The time period between the first episode and the first subsequent recurrence.	Integer (in weeks)
What was the intraocular pressure at baseline?	Intraocular pressure at baseline	Integer (mmHg)
What was the maximum number of lesions (including scars or active lesions, from both eyes) observed at baseline?	Maximum number of lesions (scars or active lesions, from both eyes) at baseline	Integer (If none, write down “0”)
What was the maximum number of lesions (including scars or active lesions, from both eyes) observed at the last follow-up?	Maximum number of lesions (scars or active lesions, from both eyes) at last follow-up.	Integer
What was the patient’s final visual acuity?	Best corrected visual acuity (BCVA) after treatment.	Snellen score.
What was the patient’s visual acuity at the time of presentation?	Best corrected visual acuity (BCVA) at presentation. BCVA defined as the best possible vision that an eye can achieve with the use of glasses or contact lenses	Snellen score.
What was the primary criterion for discontinuing systemic antibiotic therapy?	Indicates the main criterion used to decide when to stop systemic antibiotic therapy for the patient. Options include stopping therapy once the lesion is inactive, after the lesion shows a response to treatment, or based on a fixed time interval.	1 = Until the lesion is inactive (Systemic therapy was stopped when the lesion became inactive) 2 = Until the lesion shows a response to treatment (Systemic therapy was stopped once the lesion demonstrated a positive response to treatment, though it may not be fully inactive) 3 = Fixed time interval (Systemic therapy was discontinued after a pre-set duration, regardless of lesion activity status) 99 = Unknown
What was the primary source or approach used to prescribe drug treatment for the patient?	Indicates the approach or source of expertise relied upon in prescribing medication for the patient, such as whether the prescription was made independently, in consultation with an infectious disease specialist, or referred entirely for specialist treatment.	1 = Prescribed based on general ophthalmologist expertise 2 = Prescribed based on uveitis specialist expertise 3 = Prescribed as advised by an infectious disease physician 4 = Patient referred to an infectious disease physician for treatment 5 = Other approaches 99 = Unknown
What was the route of referral for the patient, and by whom was the referral made?	Indicates the pathway through which the patient sought or was referred for specialist consultation. This includes whether the referral came from an ophthalmologist or non-ophthalmologist with a preliminary diagnosis, or if the patient sought consultation independently	1 = Referred by an ophthalmologist with a diagnosis of non-specific uveitis 2 = Referred by an ophthalmologist with a diagnosis of ocular toxoplasmosis 3 = Referred by a non-ophthalmologist with a diagnosis of ocular toxoplasmosis 4 = Referred by a non-ophthalmologist with a non-specific diagnosis 5 = Self-referred without any prior diagnosis or referral 99 = Unknown
What was the treatment route administered to the patient?	The method by which treatment is administered to the patient (e.g., oral, intravenous, intravitreal), affecting the delivery and effectiveness of the medication.	1 = Systemic, 2 = Intravitreal, 3 = Both, systemic and intravitreal, 99 = Unknown
What was the **worst** degree of ac cells develop over the course of the disease?	Worst degree or severity of ac cells, using the SUN Criteria	1 = 0 (< 1 cell) 2 = 0.5+ (1–5 cells) 3 = 1+ (6–15 cells) 4 = 2+ (16–25 cells) 5 = 3+ (26–50 cells) 6 = 4+ (> 50 cells) 99 = Unknown
What was the worst degree of vitreous cells develop over the course of the disease?	Worst degree or severity of vitreous cells, using the SUN Criteria	1 = 0 (< 1 cell) 2 = 0.5+ (1–5 cells) 3 = 1+ (6–15 cells) 4 = 2+ (16–25 cells) 5 = 3+ (26–50 cells) 6 = 4+ (> 50 cells) 99 = Unknown
What were the characteristics of the lesion after treatment?	Characteristics of the lesion after treatment.	1 = Atrophic scar 2 = Pigment changes 3 = Residual inflammation 4 = Subretinal fibrosis 5 = Other 99 = Unknown
What were the OCT features observed during the active episode?	Key features of the lesion as observed through OCT imaging.	1 = Partial Retinal Infiltration, 2 = Full Thickness Retinal Infiltration, 3 = Subretinal fluid, 4 = Intraretinal Fluid, 5 = Bacilary Detachment, 6 = Paracentral Acute Middle Maculopathy, 7 = Retinal thinning, 8 = Epiretinal membrane, 9 = Vitreous Cells, 10 = Pre-retinal deposits, 11 = Other. 99 = Uknown
What were the patient’s CD4 + levels?	measurement of levels of CD4 + T Cell Lymphocites closest to the first consultation	Integer (mg/dL)
What were the patient’s IgG levels?	The concentration of Immunoglobulin G (IgG) antibodies in the patient’s blood, indicating either past exposure to an infection or a long-term immune response.	Integer (IU/ml)
What were the patient’s IgM levels?	The concentration of Immunoglobulin M (IgM) antibodies in the patient’s blood, indicating an initial or recent immune response, often to infection.	Integer (IU/ml)
What were the PCR test results from aqueous samples?	Results of PCR testing for Toxoplasma gondii DNA in ocular fluids (aqueous humour).	0 = Not done, 1 = Positive, 2 = Negative, 3 = Other 99 = Unknown
What were the PCR test results from vitreous samples?	Results of PCR testing for Toxoplasma gondii DNA in ocular fluids (vitreous).	0 = Not done, 1 = Positive, 2 = Negative, 3 = Other 99 = Unknown
What were the primary symptoms at the time of presentation?	Patient-reported primary symptom(s) at presentation.	0 = Asymptomatic 1 = Visual impairment 2 = Floaters 3 = Eye pain 4 = Redness 5 = Photophobia 6 = Scotoma 7 = Other 99 = Unknown
What were the results of the IgG serologic toxoplasmosis testing?	Results of serological tests for IgG toxoplasmosis antibodies.	0 = Not done, 1 = Positive, 2 = Negative, 3 = Other 99 = Unknown
What were the results of the IgM serologic toxoplasmosis testing?	Results of serological tests for IgM toxoplasmosis antibodies.	0 = Not done, 1 = Positive, 2 = Negative, 3 = Other 99 = Unknown
Where was the active lesion located?	The anatomical location of the active lesion within the eye based on Recommendations from the International Widefield Imaging Study Group	1 = Fovea, 2 = Posterior pole (retina within the arcades and just slightly beyond them ~ 50 degrees field of view), 3 = Mid-periphery (region of the retina up to the posterior edge of the vortex vein ampulla ~ 60 to 120 degrees field of view), 4 = Far Periphery (region of the retina anterior to the vortex vein ampulla ~ 110 to 220 degrees field of view), 5 = Juxtapapillary nasal, 6 = Juxtapapillary temporal, 7 = Optic nerve head, 8 = Other
Which eye(s) is/are affected (right, left, or both)?	Laterality of the infection.	1 = Unilateral (Right eye), 2 = Unilateral (Left eye), 3 = Bilateral

From an operational standpoint, incorporating standardized case report forms—drawing on existing frameworks such as the CDEs from the NIH/National Institute of Neurological Disorders and Stroke (NINDS)—could streamline data management and monitoring processes across different study arms [10, 19–21]. The CDEs provide a structured set of standardized definitions, formats, and protocols for collecting data, which ensures consistency and comparability across studies. For example, in traumatic brain injury research, CDEs have been instrumental in harmonizing data collection, enabling more robust data sharing and re-use, as highlighted by Hicks et al. [20] Similarly, CDEs have facilitated the systematic assessment of behavioral phenotypes in disorders of consciousness, as demonstrated by Yakhkind et al. [19] Furthermore, CDEs have been successfully adopted in assessing psychiatric comorbidities across brain disorders, underscoring their versatility and utility in diverse research contexts [21]. Following the NINDS example, the resulting set of CDEs for OT will be submitted to the NIH CDEs Repository to enhance their accessibility and findability [22]. 

Although vast research has been done on OT outcomes, leading to a better understanding of the disease [23–25], only a few studies have made their original data available. For instance, Sittivarakul et al. published their data on clinical characteristics, visual acuity outcomes, and factors associated with vision loss in patients with active OT at a Thai tertiary center [26]. While their dataset provides valuable insights into the disease’s clinical course, the absence of a standardized data dictionary limits its utility for broader integration into multicenter studies or patient-level meta-analyses. This exemplified the challenges associated with data reuse and integration in larger cohorts [27]. Without clear definitions and structured formats, reconciling variables across different datasets becomes challenging, hindering the ability to pool data or draw more robust conclusions. This underscores the importance of adopting standardized CDEs with well-defined dictionaries to ensure data consistency, interoperability, and reusability in ophthalmology research.

The interdisciplinary approach allowed for a comprehensive review of the questions to ensure clarity and ease of response and prevent ambiguities or leading statements. Additionally, response bias was mitigated by maintaining participant anonymity throughout the process, including during the analysis and the review of suggestions and changes. These measures collectively contribute to our study’s robustness and the reliability of its outcomes. These CDEs are designed to be compatible with electronic health records (EHRs) to facilitate implementation in real-world settings. They may be adapted into standardized case report forms or structured data entry templates. Once published and endorsed through the NIH Common Data Elements Repository, they will be better positioned for integration into clinical data systems and research platforms, supporting harmonization across institutions and improving data interoperability.

Despite these strengths, practical challenges remain in implementing such an extensive set. While this comprehensive pool of CDEs underscores the depth of information relevant to OT, not all variables may be feasible to collect in routine or resource-limited settings. Researchers may, therefore, need to tailor subsets of these CDEs to specific investigative goals or study designs [28]. Furthermore, the success of this endeavor requires ongoing collaboration and training across research centers to ensure adherence and uniformity in data collection. To address this, PROTON plans to pilot these CDEs in a multicenter observational study to refine them based on practical challenges and user feedback. Such a study is intended to be prospective, evaluating the impact of different treatment schemes and systemic corticosteroids on OT outcomes, including the effect of timing on disease progression and recovery.

Another consideration is the evolution of diagnostic and therapeutic modalities for OT [29]. As new technologies and treatments emerge, the relevance and perceived importance of certain CDEs may shift. Consequently, the CDE set identified here should be regarded as an adaptable resource, periodically updated in response to advancements in the field and feedback from real-world implementation. Another limitation of this study is that the study participants’ geographic distribution presents limitations due to confirmation bias. The majority of participating centres were located in South America (43%) and South Asia (30%), with limited representation from North America (3%), Europe (17%), East Asia (3%), and Oceania (3%). However, this distribution aligns with the regions where OT cases are most prevalent and where the greatest clinical needs exist, prioritizing perspectives from areas most affected by OT. Future studies should aim to include broader geographic representation, including North America, Europe, East Asia, and Oceania, in subsequent pilot or multicenter validations.

Beyond the foundational role of CDEs in standardizing data collection, these elements serve as a critical framework for enabling future research initiatives. By leveraging CDEs, researchers can design projects that integrate with existing ophthalmic and infectious disease databases, enhancing data interoperability and facilitating large-scale, multicenter analyses. Furthermore, the incorporation of omics data—such as genetic and immunological profiles—could provide deeper insights into host susceptibility and disease severity. Structured imaging annotation protocols, built upon CDEs, could also support AI-driven analyses for lesion classification, stratification, disease monitoring, and prediction of recurrence and resolution. Finally, systematic inclusion of longitudinal follow-up data will allow researchers to track recurrence rates, treatment efficacy, and disease progression over time, unlocking new avenues for understanding and managing the disease.

Moreover, expanding CDEs to encompass patient-reported outcomes (PROs) and quality-of-life (QOL) metrics will offer a more holistic assessment of disease burden, especially in chronic or recurrent cases, but it’s likely that PROs and QOLs will not need to be specific to OT and should engage a different cohort of subject matter experts and people with lived experience. Cost-effectiveness and healthcare utilization metrics should also be considered to evaluate treatment accessibility and economic impact. Pediatric-specific adaptations for congenital and early-onset OT, along with neurodevelopmental assessments, would further strengthen the applicability of CDEs across diverse patient populations. Training programs and dedicated research networks should be established to support the implementation of these CDEs, ensuring consistency, usability, and continuous refinement based on real-world feedback. Collectively, these enhancements will position OT CDEs as a dynamic and adaptable resource for advancing research, clinical care, and global collaboration in OT. To ensure broader applicability, future efforts should include the translation and cultural adaptation of CDE definitions into relevant local languages, particularly in endemic regions where OT prevalence is highest.

In conclusion, our Delphi study provides a robust starting point for a standardized OT data collection approach. These recommendations are intended to improve data interoperability, enabling data pooling to get more robust conclusions about disease progression, treatment, prophylaxis, prevention efficacy, and patient outcomes. Adopting these consensus-based CDEs can enhance comparability across studies, strengthen the evidence base for treatment strategies, and ultimately improve patient outcomes. Future endeavors should focus on practical implementation strategies, validation in varied clinical contexts, language translations, and iterative revisions to ensure this framework remains relevant and feasible.

## Supplementary Information

Supplementary Material 1.

Supplementary Material 2.

## Data Availability

All data generated or analysed during this study are included in this published article and its supplementary information files.
